# Indocyanine green fluorescence video angiography reduces vascular injury–related morbidity during micro-neurosurgical clipping of ruptured cerebral aneurysms: a retrospective observational study

**DOI:** 10.1007/s00701-019-04029-6

**Published:** 2019-09-06

**Authors:** Tamara Tajsic, James Cullen, Mathew Guilfoyle, Adel Helmy, Ramez Kirollos, Peter Kirkpatrick, Rikin Trivedi

**Affiliations:** Department of Neurosurgery, Cambridge University Hospital, Box 166, Hills Road, Cambridge, CB20QQ UK

**Keywords:** Subarachnoid haemorrhage, Cerebral aneurysm, Clipping, Indocyanine green video angiography (ICG-VA)

## Abstract

**Background:**

Specific procedural complications in aneurysm surgery are broadly related to vascular territory compromise and brain/nerve retraction; vascular complications account for about half of this. Intraoperative indocyanine green video angiography (ICG-VA) provides real-time high spatial resolution imaging of the cerebrovascular architecture, allowing immediate quality assurance of aneurysm occlusion and vessel integrity. The aim of this study was to examine whether the routine use of ICG-VA reduced early procedural complications related to vascular compromise or injury during micro-neurosurgical clipping of ruptured cerebral aneurysms.

**Methods:**

Retrospective comparative observational study of 412 adult good-grade (WFNS 1 or 2) SAH patients who had undergone microsurgical clipping without (*n* = 200, 2001–2004) or with (*n* = 212, 2009–2015) ICG-VA in a high-volume neurosurgical centre.

**Results:**

The ICG-VA group had a significantly lower incidence of procedural vascular complications (7/212; 3.3%) compared with the non-ICG-VA group (19/200; 9.5%) (Fisher’s exact test *p* = 0.0137).

**Conclusions:**

ICG-VA is a straightforward, easy-to-use, intraoperative adjunct which significantly reduces avoidable ‘technical error’ related morbidity.

## Introduction

Optimal micro-neurosurgical treatment (clipping) of intracranial aneurysms involves complete exclusion of the aneurysm from the circulation with the preservation of parent, branching and perforating vessels. Inadvertent occlusion or compromise of these vessels can produce perfusion deficits, critical ischaemia and stroke with consequent neurological deficit and direct repercussions on functional recovery. Ischaemic events are reported to complicate clipping in up to 22% of cases [1–3]. Procedural complication rates following the micro-neurosurgical treatment of unruptured intracranial aneurysms (elective clipping) are more easily quantifiable as any postoperative deficit that might develop can be attributed to the surgical procedure [1, 9, 18]. The situation is more complicated for the clipping of ruptured aneurysms (emergency clipping) as the development of post-subarachnoid haemorrhage (SAH) brain swelling, the presence of blood in the natural anatomical planes and the higher risk of intraoperative aneurysm rupture make the procedure technically more difficult thus increasing the surgical risks. In a previous study [2], we found that 9.5% (19 out of 200) of patients with World Federation of Neurosurgical Societies (WFNS) grade 1 or 2 (good-grade) SAH who underwent clipping of ruptured aneurysms (emergency clipping) between 2001 and 2004 in our neurosurgical department suffered a surgical complication due to vascular injury or compromise. This was the first study to examine the surgical complications specifically following surgery for ruptured cerebral aneurysms in good-grade SAH patients and found that, although the overall outcomes were good, majority of poor outcomes were due to surgical complications, particularly the vascular ones. These findings emphasise the utmost importance of accurate clip placement and preservation of vessel integrity in aneurysm surgery [2].

The surgical procedure itself has been refined in recent years, particularly with the use of intraoperative adjuncts to assess flow and inform about the adequacy of clip placement. Intraoperative catheter angiography (digital subtraction angiography, DSA) is still believed to be the gold standard [16]. However, this procedure increases operating time and requires hybrid operating suites that are not available in all centres. Intraoperative indocyanine green video angiography (ICG-VA) provides real-time high spatial resolution imaging of the cerebrovascular architecture, allowing immediate quality assurance of aneurysm occlusion and vessel integrity; facilitating intraoperative clip adjustment if necessary. Its ease of use, high resolution, quick image acquisition, good safety profile and low cost meant that many neurosurgical centres nowadays rely solely on ICG-VA for the evaluation of clip position [5, 7, 13, 15–17]. Although the technique has been used for more than a decade now and is widely accepted, there are no studies to date examining specifically whether ICG-VA use reduces the risk of vascular injury in cases of micro-neurosurgical clipping of ruptured cerebral aneurysms (emergency aneurysm surgery).

The aim of this study was to examine whether the routine use of ICG-VA reduces early procedural complications related to vascular compromise or injury during clipping of ruptured cerebral aneurysms.

## Methods and materials

In our institution, the ICG-VA has been in use since 2009, integrated with the Zeiss OPMI PENTERO microscope (Carl Zeiss, Oberkochen, Germany). Prior to this, intraoperative microscopic inspection and micro-Doppler probes were utilised to assess clip position and branch vessel patency and flow. We retrospectively reviewed medical records and imaging from 212 patients who underwent emergency microsurgical clipping of cerebral aneurysms with ICG-VA following World Federation of Neurosurgical Societies (WFNS) grade 1 or 2 aneurysmal subarachnoid haemorrhage between 2009 and 2015 in our Department of Neurosurgery, Cambridge University Hospital, Cambridge, UK. We re-examined the medical records and imaging of the historical cohort of 200 patients with WFNS 1or 2 SAH who underwent micro-neurosurgical clipping of ruptured aneurysms without ICG-VA between 2001 and 2004 in the same neurosurgical department, published by Boulters and colleagues [2]. Good-grade (WFNS 1 and 2) aneurysmal SAH patients, by definition, do not have any focal neurological deficit at presentation. Patients with higher WFNS grades (3–5), delay to clipping of more than 21 days and use of non-clipping techniques (such as EC-IC bypass) were excluded, matching the inclusion and exclusion criteria set out by Boulters and colleagues [2]. The diagnosis of SAH was based on clinical presentation and computed tomography (CT) head imaging or lumbar puncture findings. The presence of an aneurysm was determined using high-resolution CT head angiography or 6-vessel catheter angiography (digital subtraction angiography; DSA). All patients were treated (i.e. aneurysms surgeries were performed) by the three senior authors of this manuscript (PK, RK, RT).

We reviewed medical records, operation (case) notes and imaging, and collected the following data: age, sex, time of ictus, WFNS score, location, type and size of aneurysm, time of surgery, the use of ICG-VA, intraoperative complications, pre- and postoperative neurological status (Glasgow coma score (GCS), focal neurological deficit (weakness and dysphasia), cranial nerve deficits) and postoperative imaging findings.

A surgical vascular complication was defined as new neurological deficit which emerged in the first 24 postoperative hours and/or a new infarct demonstrated on postoperative imaging that was consistent with aneurysmal vascular territory in the absence of confounding factors such as direct brain damage, postoperative haematoma, vasospasm or hydrocephalus. In the absence of radiological evidence, a vascular injury was recorded as any new, permanent neurological deficit with no other likely pathological mechanism. This was judged by an independent researcher and a neurosurgeon. Above interpretation is in keeping with the definition of intervention-related complications used in the recent Cochrane review by Lindgren and colleagues [14].

## Results

Our review of medical records identified 212 patients with WFNS 1 or 2 aSAH who had emergency aneurysm surgery with intraoperative ICG-VA (ICG group) between 2009 and 2015. The control group was a historical cohort of 200 patients with WFNS 1 or 2 aSAH who underwent emergency aneurysm surgery between 2001 and 2004 before ICG-VA was introduced (pre-ICG group).

The ICG and the pre-ICG groups had similar mean age (53.5 vs 51.8 years, *p* = 0.15) and age range (21–80 vs 17–82) with a similar proportion of WFNS grade 1 and grade 2 patients. The male to female ratio differed between the groups with the ICG group having a greater proportion of female patients (*p* = 0.03) (Table 1). No complications from ICG use were identified.

**Table 1:** Patient demographics. Comparison between the groups was made using Fisher’s exact test, *p* values presented

	Pre-ICG group	ICG group	
Number of patients	200	212	
Age			
Mean (SD)	51.8 (12.2)	53.4 (11.6)	*p* = 0.15
Range	17–82	21–80	
Sex			
Male	82 (41%)	65 (31%)	*p* = 0.03
Female	118 (59%)	147 (69%)	
WFNS grade			
1	152 (72%)	145 (68%)	*p* = 0.1
2	48 (24%)	67 (32%)	
Time from ictus to surgery			
Median	3	2	
Range	0–21	0–21	

With regard to aneurysm location, the ICG group involved a significantly higher proportion of patients with middle cerebral artery (MCA) aneurysms and significantly lower proportion of patients with anterior communicating artery (ACoA) aneurysms (Table 2) compared with the pre-ICG group. The relative proportion of anterior cerebral artery (ACA) aneurysms (including distal ACA aneurysms), posterior communicating artery (PCoA) and ICA aneurysms was comparable.

**Table 2:** Aneurysm location distribution in the two patient groups. Comparison between the groups was made using Fisher’s exact test

Aneurysm location	Pre-ICG group	ICG group	
Anterior communicating a.	81 (40.5%)	55 (26.0%)	*p* = 0.001
Anterior cerebral a.	3 (1.5%)	8 (3.7%)	*p* = 0.339
Middle cerebral a.	48 (24.0%)	85 (40.1%)	*p* = 0.005
Posterior communicating a.	49 (24.5%)	47 (22.2%)	*p* = 0.641
Internal carotid a.	12 (6.0%)	17 (8.0%)	*p* = 0.553
Posterior inferior cerebellar a.	5 (2.5%)	0	
Basilar a.	2 (1.0%)	0	

Next, we compared the rate of procedural vascular errors between the two groups and found that the use of ICG-VA significantly reduced the overall rate of procedural vascular complications: 3.3% when ICG-VA was used compared with 9.5% without the use of ICG-VA (Table 3).

**Table 3:** Vascular procedural complication rates. Comparison between groups made using Fisher’s exact test, *p* values presented for each group comparison

	Pre-ICG group	ICG group	
Vascular complications	19/200 (9.5%)	7/212 (3.3%)	*p* = 0.014
Vascular complications per aneurysm location			
Anterior communicating a.	6	1	
Middle cerebral a.	8	3	
Posterior communicating a.	0	2	
Internal carotid a.	5	1	

**Fig. 1:**
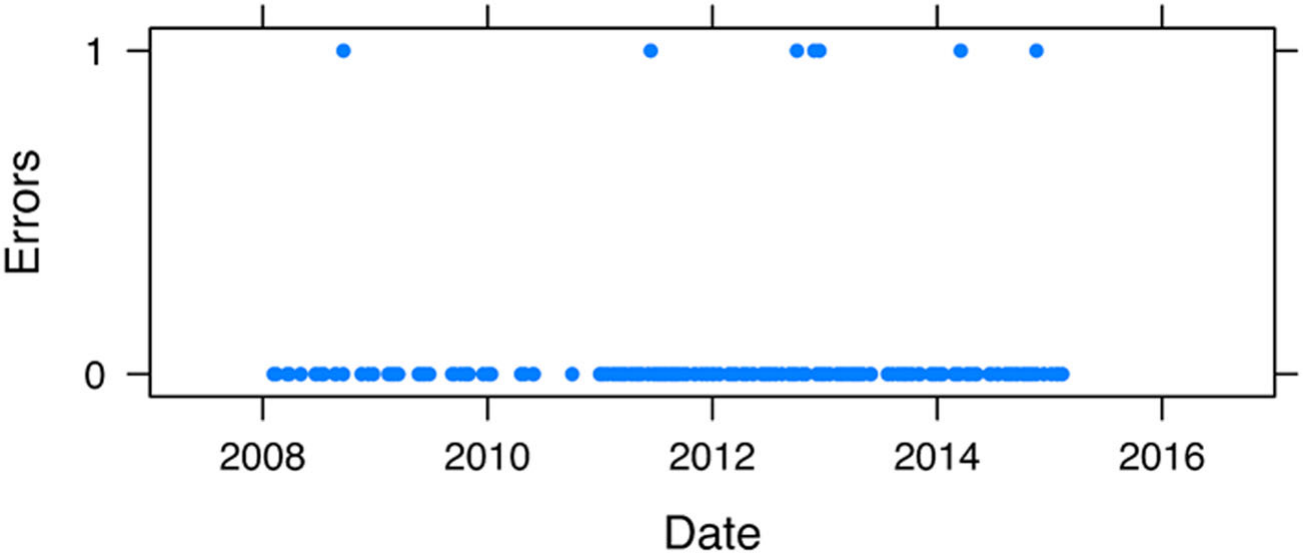
Even distribution of cases with vascular procedural complications over time suggests that there is no learning curve with ICG-VA

Interestingly, the procedural errors were distributed evenly over time indicating that there is no learning curve associated with the use of ICG-VA (Fig. 1).

## Discussion

Complications arising from procedural errors are significant, yet potentially modifiable contributors to postoperative morbidity and mortality following open intracranial aneurysm surgery. Inadvertent vessel occlusion with consequent intraoperative and postoperative ischaemic complications can be detected in up to a quarter of cases of aneurysm clipping negatively affecting clinical outcomes [1–3, 5, 8, 13]. Prompt detection of vessel compromise and immediate correction of clip position are fundamental in preventing ischaemic complications. Out of the intraoperative adjuncts available, intraoperative DSA is still considered the gold standard [4, 10, 11, 13, 16]. A recent metanalysis shows that intra- or postoperative DSA detected mis-clippings in 4.5% of the cases of ICG-VA-supported surgeries, just over half of those were cases of vessel compromise [16]. In the study by Lai and Morgan, ICG-VA was found to be critical, and superior to intraoperative DSA, in assessing the patency of small perforators [13]. Intraoperative DSA requires hybrid theatres, extends operative time, and is, overall, more expensive and carries a 0.4–2.6% risk of complications [4, 10, 11]. ICG-VA is quick, safe, easy to use and cheap, making it far more widely utilised than intraoperative DSA. Although it cannot completely replace intraoperative DSA, ICG-VA is a reasonable and reliable alternative [13, 15, 16]. This study shows that using intraoperative ICG-VA during clipping of ruptured cerebral aneurysms significantly reduces the risk of procedural vascular complications pertaining to suboptimal clip placement (from 9.5 to 3.3%), providing an important contribution to the existing knowledge base on the efficiency and efficacy of ICG-VA.

In this study, we included patients who presented with ‘good grade’ (WFNS 1 and 2) aneurysmal subarachnoid haemorrhage and the rationale for this was twofold. Firstly, this is the predominant patient group in which sound surgical technique is paramount in achieving a good outcome [2]. When compared with surgery for non-ruptured aneurysms, the vulnerable brain following SAH is less tolerant to retraction injury, transient ischaemia and complete or even partial vascular compromise resulting in suboptimal blood flow [2, 8]. Secondly, WFNS grades 1 and 2 patients, by definition, do not have any focal neurological deficit. This makes postsurgical ischaemic complications attributable directly to intraoperative mishaps more readily identified. The two patient groups in our study had comparable numbers of WFNS 1 and WFNS 2 patients.

Like other reports [12, 13], including the recent Cochrane review on treatment modalities for patients with aneurysmal SAH [14], clinical deterioration observed during the procedure or in the first 24 h after the procedure was used as the definition of a procedure-related complication. The time of onset of the resulting deficit and the location of the vascular territory involved were then used to allocate the deficit to a technical complication. The detected effect may be even greater as there may be clinically silent infarcts not identified in either one of the groups. The development of delayed ischaemic neurological deficit (DIND) following aneurysmal SAH is multifactorial. It is possible that a suboptimally placed aneurysm clip with partial compromise of blood flow may contribute to the development of DIND which could, in turn, affect the overall patient outcome [2, 5, 7, 8, 13]. Thus, by reducing the rates of vessel compromise or occlusion, the effect on patient outcomes could be even greater.

All 412 patients included in this study were treated by dedicated neurovascular surgeons. The time period spanned in this study may raise concern that the reduced incidence of vascular complications in the latter (ICG) group may simply reflect the increasing experience of the surgeons involved. However, even at the time of performing surgery for the pre-ICG group of patients, they were already past their learning curve, each with at least 300 operative aneurysm cases before the start of the patient capture in 2001. Additionally, with the maturation of the endovascular services, the ICG group included more technically challenging cases that were not readily amenable to endovascular treatment.

This study did not specifically report on the number of cases where information derived from ICG-VA resulted in clip adjustment as it was felt that reporting the clinical result and the resulting clinically significant ischaemic deficits is by far more relevant. Although the detected vascular errors were evenly spread over time, we feel that the educational aspect from ICG utilisation was influential in reducing the ischaemic complications. Routine use of ICG-VA provided an additional unquantifiable indirect influence on the observational pattern recognition. This experience informed subsequent technical modifications in turn reducing the rate of procedural complications. With the continuous development of endovascular services, the ratio of different aneurysms undergoing micro-neurosurgical treatment changed. Largely, these represent aneurysms not amenable to endovascular treatment and can be technically challenging. As previously demonstrated in our initial series [2], the recurrent pattern was the compromise of the contralateral A2 and one of the M2 branches from clipping of ACoA and MCA aneurysms respectively. The MCA aneurysms constitute about 40% of clipped aneurysms in our ICG series; hence, the impact of the ICG-VA effect will be most apparent in this aneurysm group. These aneurysms are often wide necked and can be large and quite complex (bilobed and arising from the bifurcation) so different surgical techniques evolved over time to ensure safe clipping. The technique of ‘clip reconstruction’ for the often relatively wide-necked MCA aneurysms, which refers to the use of multiple clips of different configurations [6], was much more applied in the second group. During intra-operative ICG-VA, it was often observed that clipping the whole or the majority of the neck using one clip resulted in kinking or compromise of an M2 branch. Hence, the use of multiple clips to reconstruct the MCA aneurysm neck and preservation of the M2 branches was more readily used in the more recent group as a result of the information obtained by using ICG-VA in the initial cases.

In summary, ICG-VA is a straightforward and commonly used surgical adjunct for aneurysm clipping which significantly reduces the incidence of vascular technical errors, particularly in the clipping of MCA aneurysms. These reductions in surgical complications may lead to reduced morbidity postoperatively in patients with aneurysmal SAH and should be taken into consideration when deciding between endovascular and micro-neurosurgical treatment for ruptured cerebral aneurysms.
